# European Epidemiological Patterns of Cannabis- and Substance-Related Body Wall Congenital Anomalies: Geospatiotemporal and Causal Inferential Study

**DOI:** 10.3390/ijerph19159027

**Published:** 2022-07-25

**Authors:** Albert Stuart Reece, Gary Kenneth Hulse

**Affiliations:** 1 Division of Psychiatry, University of Western Australia, Crawley, WA 6009, Australia; gary.hulse@uwa.edu.au; 2 School of Medical and Health Sciences, Edith Cowan University, Joondalup, WA 6027, Australia

**Keywords:** cannabis, cannabinoid, genotoxicity, epigenotoxicity, transgenerational inheritance

## Abstract

As body wall congenital anomalies (BWCAs) have a long history of being associated with prenatal or community cannabis exposure (CCE), it was of interest to investigate these epidemiological relationships in Europe given the recent increases in cannabis use prevalence, daily intensity, and Δ9-tetrahydrocannabinol (THC) potency. Methods: This study makes use of BWCA data from Eurocat, drug exposure data from the European Monitoring Centre for Drugs and Drug Addiction, and income from the World Bank. Results: The mapping analysis showed that BWCARs increased in France, Spain, and the Netherlands. The bivariate mapping analysis showed that the BWCA rates (BWCAR) and the cannabis resin THC concentration rose simultaneously in France, the Netherlands, Bulgaria, Sweden, and Norway. The bivariate ranking of the BWCARs by median minimum E-value (mEV) was omphalocele > diaphragmatic hernia > abdominal wall defects > gastroschisis. With inverse probability weighted multivariable panel regression, the series of BWCAs, including gastroschisis, omphalocele, and diaphragmatic hernia, was positively related to various metrics of cannabis use from *p* = 2.45 × 10^−14^, 4.77 × 10^−7^ and <2.2 × 10^−16^. With geospatial regression, the same series of BWCAs was related to cannabis metrics from *p* = 0.0016, 5.28 × 10^−6^ and 4.88 × 10^−9^. Seventeen out of twenty-eight (60.7%) of the E-value estimates were >9 (high range), as were 14/28 (50.0%) of the mEVs. Conclusion: The data confirm the close relationship of the BWCARs with the metrics of CCE, fulfill the quantitative criteria of causal inference, and underscore the salience of the public health impacts of cannabinoid teratogenicity. Of major concern is the rising CCE impacting exponential cannabinoid genotoxic dose-response relationships. CCE should be carefully restricted to protect the food chain, the genome, and the epigenome of coming generations.

## 1. Introduction

The relationship between body wall congenital anomalies (BWCAs) and cannabis exposure has been documented by researchers over many years. The relationship between omphalocele, a condition whereby the rapid elongation of the gut and increased liver size in the early stage of gestation reduces intra-abdominal space and forces the intestinal loops or other internal organs to protrude outside out of the abdominal cavity, and cannabis exposure was initially identified in animals [1,2,3] and has since been noted in several human studies [4,5,6,7,8]. Gastroschisis has been linked with cannabis use by many series [9,10,11,12,13,14,15], and in several large national and state-level epidemiological series [4,6,7,11,16]. Diaphragmatic hernia has been linked with cannabis exposure in the USA by researchers from the Center for Disease Control [17] and more recently with patterns of US cannabidiol exposure [6].

Interestingly, based on the association of several vasoactive drugs with elevated incidences of gastroschisis, this anomaly has been linked with vascular compromise as a possible cause [15,18,19].

Cannabinoid teratogenicity is a subset of cannabinoid genotoxicity, which also includes cannabinoid cancerogenicity and cannabinoid-induced accelerated aging. It is therefore important to read the present report in the light of this wider epidemiological and mechanistic literature.

Multiple pathways to cannabinoid genotoxicity have been described, some of them for many decades, including the induction of severe dysmorphology of sperm [20,21]; the severe inhibition of oocyte division [22]; single- and double-stranded DNA breaks; chromosomal breakages [23,24,25]; and end-to-end fusions, including ring and chain translocations [21]; the oxidation of DNA bases [23]; interference with sperm histone-protamine exchange [26]; the inhibition of oviduct function [27]; the inhibition of Sertoli cell function [27]; the reduction in DNA, RNA, and protein synthesis [28,29,30,31,32,33]; the alteration of DNA methylation patterns [34,35,36,37,38,39,40,41,42]; the reduction in histone synthesis [26]; the reduction in phospho- and acetylated-activated histone production [29,30,43]; and the heritable passage of altered patterns of DNA and histone expression to subsequent generations [34,35,36,37,38,39,40,41,42,44].

Most recently, a series of fascinating epigenomic studies have been performed in rats and humans on both cannabis dependence and cannabis withdrawal; these have returned a very valuable and intriguing wealth of data in relation to the disruption of pathways in embryonic morphogenesis and in cannabis-related tumorigenesis [40,41,42]. Some of these fascinating and important data are considered further in Section 4.

Recent reports from Europe indicate a dramatic rise in community exposure to cannabinoids in some European countries. In these jurisdictions, the three important trends of increased prevalence of use, rising intensity of daily use, and the rising Δ9-tetrahydrocannabinol (THC) potency of available cannabis products acting together have dramatically increased the population exposed to cannabinoids [45,46], a feature importantly exacerbated by the very long half-life of lipid-soluble cannabinoids in adipose, brain, and gonadal fat deposits.

Cannabinoids have a well-described exponential genotoxic dose-response curve, which has been found both with direct mutagenic assays [23,47,48,49,50,51,52,53] and in relation to mitochondrial intermediary metabolism [54,55,56,57,58,59], on which many genomic and epigenomic reactions are based as they supply energy and substrates to key nuclear reactions [60].

Dramatically rising cannabinoid exposure, as observed in some European countries, which takes the community exposure across the threshold of the higher-order genotoxicity reactions, would be expected to manifest as a relatively abrupt rise in major genotoxic outcomes. Indeed, there is some concern that this may be happening in parts of rural France where in areas sown with large cannabis crops both calves and human babies are being born limbless at sixty times the historical rate [61,62,63]. The possibility that cannabinoids may have entered the food chain either through the water table or stock feed has not been discounted. Similar reports have recently also come from parts of rural Germany [64]. Conversely, in nearby Switzerland, where cannabinoids are not permitted in the food chain, no such outbreak is reported.

For all of these reasons, it was apparent that an epidemiological study of BWCA rates (BWCARs) in modern Europe was timely, interesting, and of considerable public health importance. The pre-determined analytical plan was to conduct bivariate analyses and multivariable analysis in a causal inferential paradigm and also to consider the analysis in the native space–time environment from which the data derive to allow optimal handling of several technical analytical factors.

## 2. Methods

### 2.1. Data

The data on all the available congenital anomaly rates were downloaded by each individual year for each of the 14 nations from the European Network of Population-Based Registries for the Epidemiological Surveillance of Congenital Anomalies (EUROCAT) website [65] and analyzed. The EUROCAT total congenital anomaly rate includes anomaly rates amongst live births, stillbirths, and cases where early termination for anomaly was carried out; all of these are combined together so that they represent a total overall picture across all classes of births. The nations selected were chosen on the basis of the availability of their congenital anomaly data for most of the years from 2010–2019. National tobacco (percentage of daily tobacco use prevalence) and alcohol (litres of pure alcohol consumed per capita annually) use data were downloaded from the World Health Organization [66]. Drug use data for cannabis, amphetamines, and cocaine were taken from the European Monitoring Centre for Drugs and Drug Addiction (EMCDDA) [67]. Last month’s cannabis use data were also supplemented by the data on the tetrahydrocannabinol (THC) content of cannabis herb and resin provided in the recently published reports [46]. Data on daily cannabis use were also available from the EMCDDA and were collated in recent reports [46]. Median household income data (in USD) were taken from the World Bank [68].

#### 2.1.1. Derived Data

The availability of several metrics of cannabis use, exposure, and consumption made it possible to calculate various derived metrics. Hence, last month’s cannabis use prevalence data were multiplied by the THC content of cannabis herb and resin in order to derive compound metrics. These metrics were also multiplied by the imputed daily cannabis use prevalence rates to derive further compound metrics for both cannabis herb and resin.

#### 2.1.2. Data Imputation

The missing data were completed by linear interpolation. This was particularly the case for daily cannabis use. Fifty-nine data points on daily cannabis use from the EMCDDA were available for these 14 nations across this period. Linear interpolation expanded this dataset to 129 datapoints (further details are provided in Section 3). Data on the cannabis resin THC concentration were not available for Sweden. However, it was noted that the resin-to-herb THC concentration was almost constant in nearby Norway at 17.7; so, this ratio was applied to the Swedish cannabis herb THC concentration data to derive estimates of the Swedish cannabis resin THC concentration. Similarly, data for the cannabis resin THC concentration in Poland were not available. The resin-to-herb THC concentration ratio of nearby Germany was used to estimate the resin THC content in Poland from the known Polish herb THC concentrations. As geospatial analytical techniques do not tolerate missing data, the dataset was completed by the last observation carried forwards or backwards for Croatia in 2018 and 2019 and for the Netherlands in 2010. It was not appropriate to use multiple imputation methods for this dataset as multiple imputation cannot be applied in panel or spatial multivariable regression techniques.

### 2.2. Statistics

The data were processed in R Studio version 1.4.1717 (R Foundation for Statistical Computing, R Core Development Team, Chicago, IL, USA) based on R version 4.1.1 from the Comprehensive R Archive Network and the R Foundation for Statistical Computing [69]. The analysis was conducted in December 2021. The data were manipulated using dplyr from the tidyverse [70]. The data were log transformed where appropriate to improve compliance with normality assumptions based on the results of a Shapiro–Wilk test. The graphs were drawn in ggplot2 from tidyverse. The maps were drawn using ggplot2, sf (simple features) [71], and both the custom color palettes and the palettes were taken from the viridis and viridisLite packages [72].

Bivariate maps were drawn with the package colorplaner [73]. All the illustrations are original and have not been published before. Linear regression was conducted in Base R. Mixed effects regression was performed using the package nlme [74]. In all the multivariable models, the model reduction was by the classical technique of serial deletion of the least significant term to yield a final reduced model, which is the model presented. Multiple linear models were processed in a single pass using combined techniques from the R packages purrr and broom [70,75,76]. The overall effect of the covariates in multivariable models may be quantified as the marginal effect. In this case, the overall marginal effect was calculated using the R package margins [77].

### 2.3. Covariate Selection

The presence of multiple different metrics for cannabis consumption and exposure created a problem for analysis as it was not clear which was the most appropriate metric to employ for any particular model. Indiscriminate use of excessive covariates in a multivariable model would unnecessarily consume degrees of freedom and thereby restrict the ability to assess interactions. This issue was formally addressed by the use of random forest regression using the R package ranger [78], with variable importance being formally assessed via the R package vip (variable importance plot) [79]. The most predictive covariates from this process were entered into the regression modelling equations. The tables from this analysis are presented in Section 3.

### 2.4. Panel and Geospatial Analysis

Panel analysis was conducted using R package plm [80] across both space and time simultaneously, using the “twoways” effect. The spatial weights matrix was calculated using the edge and corner “queen” relationships, the using R package spdep (spatial dependency) [81]. Geospatial modelling was conducted using the spatial panel random effects maximum likelihood (spreml) function from the package spml, which allows detailed modelling and correction of model error structures [82,83]. Such models may produce four model coefficients of interest, which are useful in determining the most appropriate error structure for the model. These coefficients are phi, the random error effect; psi, the serial correlation effect; rho, the spatial coefficient; and theta, the spatial autocorrelation coefficient. In each case, the most appropriate error structure was chosen for each spatial model, generally taking care to preserve the model error specification across related models. The appropriate error structure was determined by the backwards methods, from the full general model to the most specific model, as has been described [84]. Both the panel and the geospatial models were temporally lagged as indicated by one to two years.

### 2.5. Causal Inference

The formal tools of causal inference were used in this analysis. Inverse probability weighting (ipw) is the technique of choice to convert a purely observational study into a pseudo-randomized study from which it is appropriate to make causal inference [85]. All the multivariable panel models presented herein were inverse probability weighted. The inverse probability weighting was performed using the R package ipw. Similarly, the E-values (expected values) quantify the correlation required of a hypothetical unmeasured confounder covariate with both the exposure of concern and the outcome of interest in order to explain away an apparently causal relationship [86,87,88]. It therefore provides a quantitative measure of the robustness of the model to extraneous covariates which have not been accounted for within the measured parameters. E-Values have a confidence interval associated with them, and the 95% lower bound of this confidence interval is reported herein. E-Value estimates greater than 1.25 are said to indicate causality [89], with E-values greater than nine being described as high [90]. The E-values were calculated from the R package E-Value [91]. Both the inverse probability weighting and the E-values are foundational and pivotal techniques used in formal causal inferential methods in order to allow causal relationships to be assessed from real-world observational studies.

### 2.6. Data Availability

Raw datasets, including 3800 lines of computation code in R, have been made freely available through the Mendeley data repository at the following URLs: https://doi.org/10.17632/tysn37t426.1 and https://doi.org/10.17632/vd6mt5r5jm.1 (accessed on 10 January 2022).

## 3. Results

Supplementary Table S1 sets out the overall details of the fourteen European nations and the four CAs contributing to this dataset. Overall, 488 data points were retrieved, representing 122 datapoints in each of the four anomalies: abdominal wall defects, diaphragmatic hernia, gastroschisis, and omphalocele. Details for other drug exposures, including compound metrics for cannabis exposure, and median household income are as indicated in the table.

The raw data for the daily cannabis use are shown in Supplementary Table S2. Many missing data points are apparent. For this reason, 70 additional points were added to this collection by linear interpolation to allow analysis of this important dataset (Supplementary Table S3).

**Figure 1:**
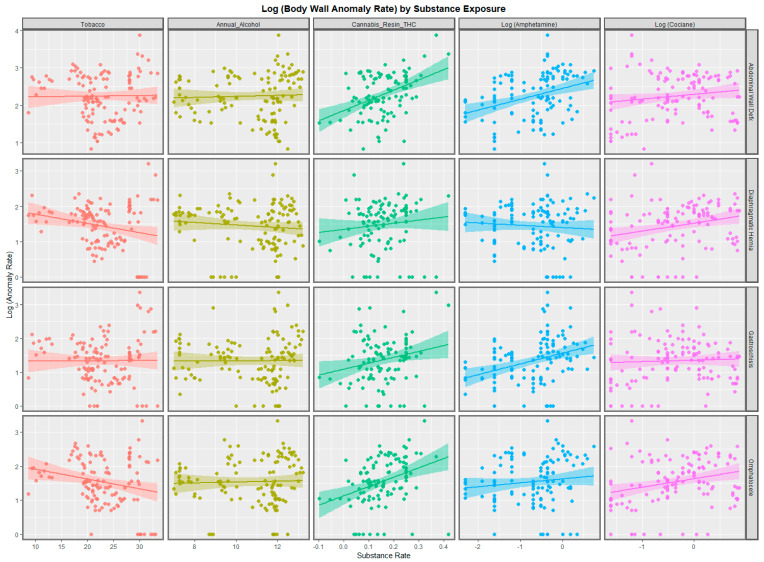
Paneled scatterplots of body wall anomalies by anomaly and by substance.

Figure 1 illustrates the bivariate relationship between tobacco, alcohol, cannabis resin THC concentration, amphetamine use, and cocaine exposures. Tobacco exposure is unrelated or negatively related to these CAs. Alcohol shows no particular relationship. Amphetamine exposure is strongly related to abdominal wall defects and gastroschisis. Cocaine is related to diaphragmatic hernia and omphalocele. Cannabis resin THC concentration is strongly related to abdominal wall defects, gastroschisis, and omphalocele.

**Figure 2:**
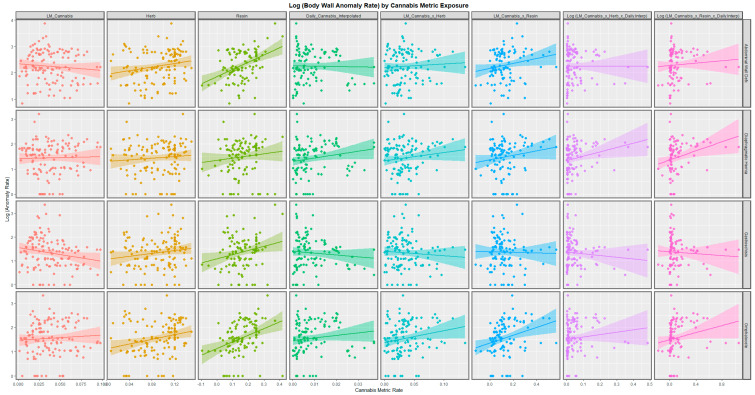
Paneled scatterplots of body wall anomalies by anomaly and by cannabis metric.

Figure 2 shows the relationship of these anomalies to the various metrics of cannabis exposure. Cannabis resin THC concentration is the most strongly positively related of all of these different parameters of cannabis exposure.

**Figure 3:**
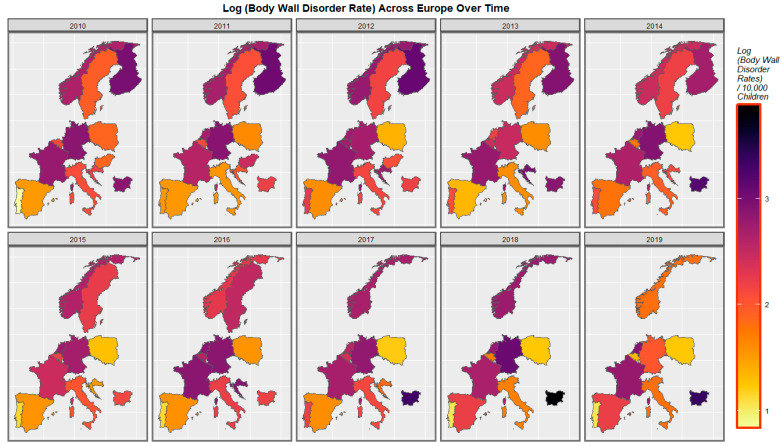
Sequential map-graph of body wall anomalies across selected European nations for each year of 2010–2019.

Figure 3 shows the spatiotemporal distribution of body wall defects across Europe over the last decade. The rates are noted to have increased in Spain, Portugal, Bulgaria and the Netherlands but decreased in Germany, Poland, and Norway.

**Figure 4:**
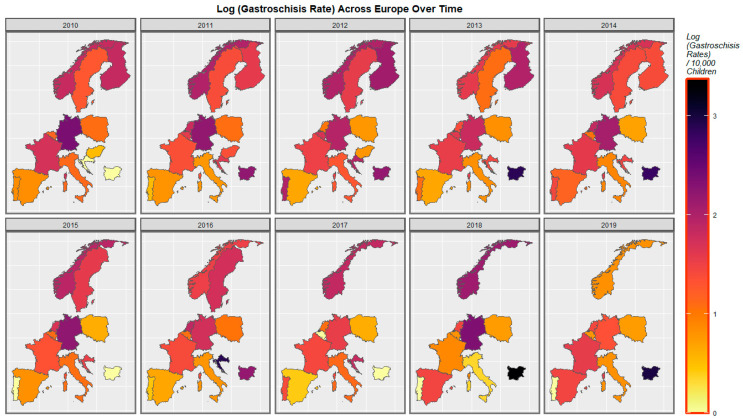
Sequential map-graph of gastroschisis across selected European nations for each year of 2010–2019.

The gastroschisis rate is noted to have declined in Germany and Norway but increased in Spain, Croatia, and Bulgaria (Figure 4).

**Figure 5:**
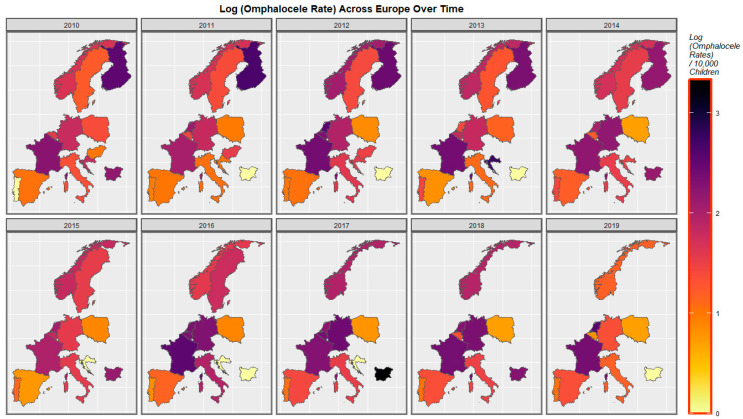
Sequential map-graph of omphalocele across selected European nations for each year of 2010–2019.

The rate of omphalocele has increased in Spain, France, and the Netherlands but declined in Germany, Poland, and Norway (Figure 5).

**Figure 6:**
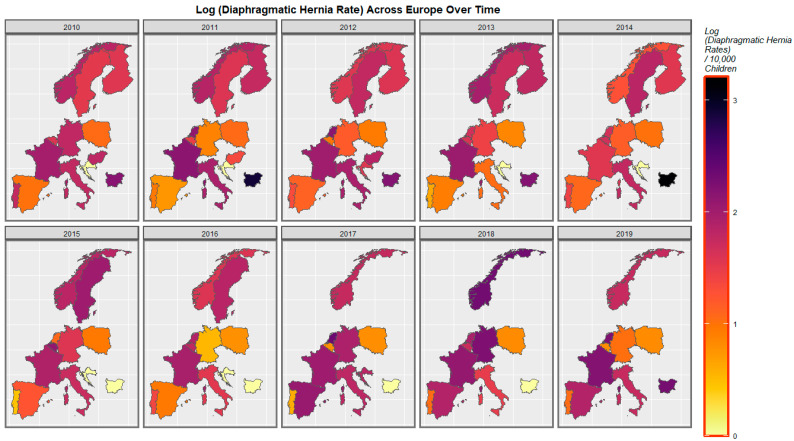
Sequential map-graph of diaphragmatic hernia across selected European nations for each year of 2010–2019.

The rate of diaphragmatic hernia increased in Spain and France across this period and fluctuated elsewhere (Figure 6).

**Figure 7:**
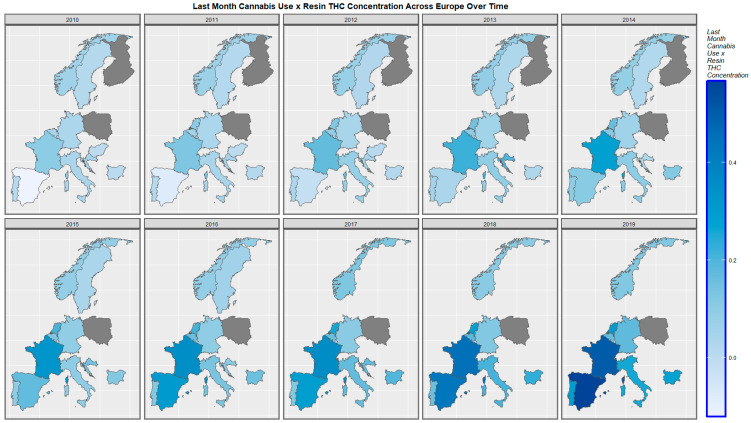
Sequential map-graph of last month’s cannabis use: cannabis resin THC concentration across selected European nations for each year of 2010–2019.

Figure 7 shows graphically the distribution of the compound cannabis metric of last month’s cannabis use: cannabis resin THC concentration. Whilst it increased across the continent, this rise was most pronounced in Spain, France, and the Netherlands.

**Figure 8:**
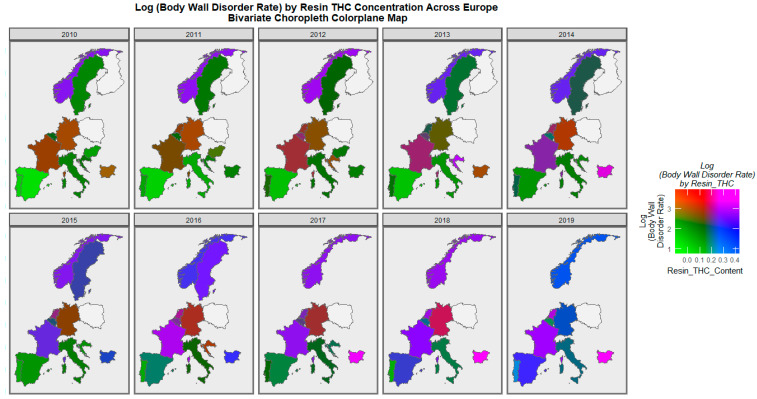
Bivariate colorplaner sequential map-graph of body wall anomalies by cannabis resin THC concentration across selected European nations for each year of 2010–2019. For explanation, please see text.

Figure 8 is a bivariate map graph which illustrates the bivariate relationship of body wall defects and cannabis resin THC concentration across Europe over the decade. One reads the map by noting where the areas of purple and pink appear; these indicate simultaneously elevated rates. The emergence of these pink and purple areas in France and Bulgaria, indicating increasing bivariate exposure, are notable in Figure 8. The area of Norway was purple throughout most of this decadal period. The Netherlands is purple from 2013–2019, and Belgium is shaded purple from 2016–2017.

**Figure 9:**
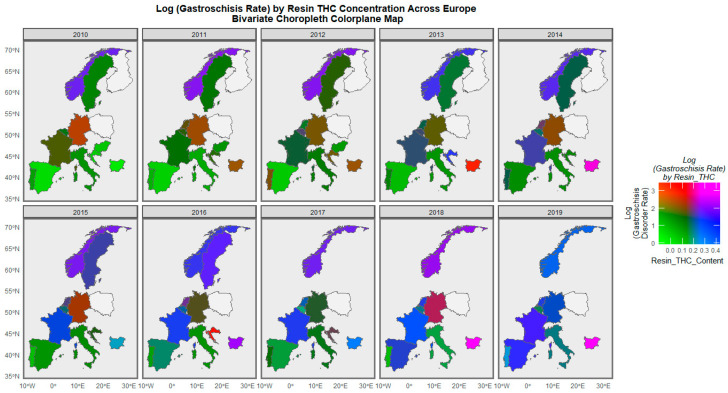
Bivariate colorplaner sequential map-graph of gastroschisis by cannabis resin THC concentration across selected European nations for each year of 2010–2019.

When the gastroschisis rate is considered against the resin THC concentration, Bulgaria is noted to turn from green to violet to purple to pink across the decade (Figure 9). Norway is shaded purple in several years. The Netherlands is shaded purple in 2014 and 2016.

**Figure 10:**
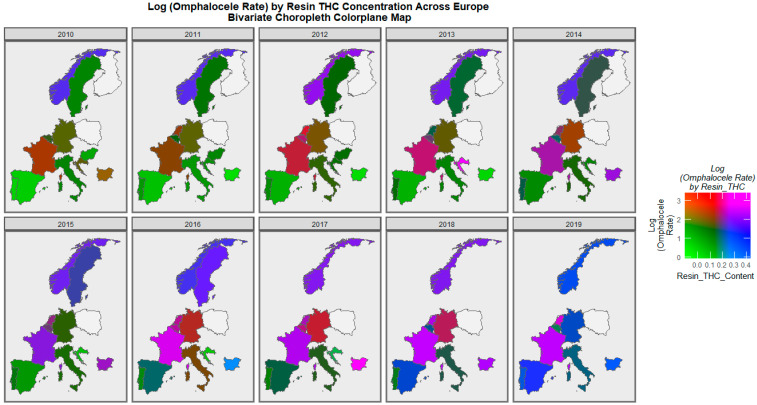
Bivariate colorplaner sequential map-graph of omphalocele by cannabis resin THC concentration across selected European nations for each year of 2010–2019.

Consideration of the omphalocele rate against the cannabis resin THC concentration reveals the pattern shown in Figure 10. France, the Netherlands, Bulgaria, and Norway turn purple or pink towards the end of the decade.

**Figure 11:**
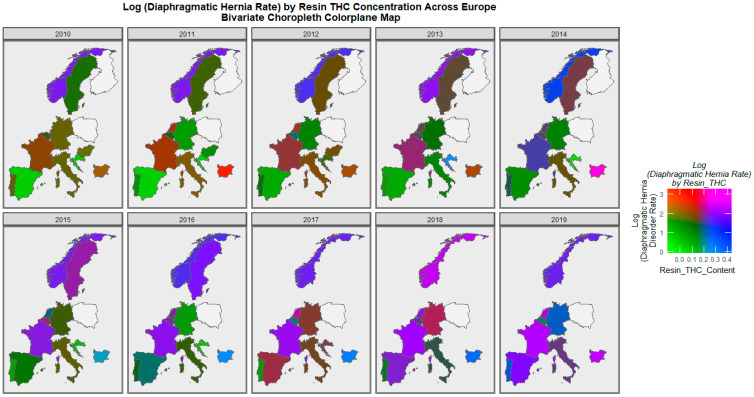
Bivariate colorplaner sequential map-graph of diaphragmatic hernia by cannabis resin THC concentration across selected European nations for each year of 2010–2019.

When diaphragmatic hernia is considered, France, Norway, the Netherlands, and Bulgaria are noted to turn purple across this period (Figure 11).

The 48 regression slopes illustrated in Figure 1 and Figure 2 can be listed in serial linear regression models (analyzed through a combined purrr-broom workflow), as shown in Supplementary Table S4. From this group, 14 regression equations may be extracted as having both positive regression coefficients and statistically significant *p*-values (Table 1). The terms in the table are listed in descending order of their minimum E-Value. It is of interest that the table is headed up by abdominal wall defects and omphalocele and terms relating to cannabis resin.

The next logical step is to move to multiple regression. However, given the finite size of the datasets and the large number of potential covariates for substance exposure, it was not immediately clear which selection of independent variables would be the most appropriate for each CA.

This matter was addressed by the use of random forest regression in tandem with variable importance calculations to derive a table of the most significant covariates for each CA. These tables are shown in Supplementary Tables S5–S7.

Supplementary Table S8 shows a series of inverse probability weighted panel regression models, including additive, interactive, and lagged models. Inverse probability weighting is important as it allows the analysis to move from a simply observational context into a pseudo-randomized paradigm from which causal inferences may properly be drawn. In all three models, the regression coefficients for the cannabis metrics are positive and highly statistically significant and range from 2.45 × 10^−14^.

Similar observations can be made about the series of inverse probability weighted panel regression models presented for omphalocele (Supplementary Table S9) and diaphragmatic hernia (Supplementary Table S10).

The next issue relates to the multivariable analysis of these data in their native space–time context, where one is able to formally and carefully control for important methodological issues, such as random effects, serial correlation, spatial correlation, and spatial autocorrelation. Supplementary Figure S1 presents a graphical illustration of the derived, edited, and final geospatial links between the nations for these data, from which the sparse spatial weight matrix was derived for spatiotemporal regression.

Table 2 presents the results of the geospatial regression for gastroschisis for the additive, interactive, and temporally lagged models. In each case, the terms including cannabis exposure metrics are included in the final regression models, have positive regression coefficients, and are statistically significant.

Similar observations apply to the series of geospatial models presented for omphalocele (Table 3) and diaphragmatic hernia (Table 4).

The E-values are applicable to each of these regression terms and coefficients. They are therefore extracted from the panel models (Table 5) and the geospatial models (Table 6). Table 7 lists these 28 E-value pairs in descending order of minimum E-value (mEV). It is noted that gastroschisis heads up this table, and the terms in the cannabis herb THC concentration comprise most of the terms near the top of the table.

These E-values may be listed sequentially, as shown in Table 8. From this list, it is noted that 17 of the 28 (60.7%) E-value estimates exceed 9 and are therefore in the high range [90], and all 28/28 (100%) exceed the threshold for causality of 1.25 [89]. For the mEVs, 14/28 (50%) are greater than 9, and all 28 (100%) exceed the threshold for causality. In each case, the media and interquartile ranges for the E-value estimates were (30.22, IQR 3.38–7.48 × 10^21^) and for mEV (10.92, IQR 1.55–9.25 × 10^11^), which show that most of the E-value estimates were in the moderate to high range.

The E-value table may be listed by anomaly, as shown in Table 9. These may then be summarized, as indicated in Table 10. The terms in this table are listed in descending order of median minimum E-value. Omphalocele is noted for being at the head of this list.

Table 7 may also be ordered by the regression term. This has been conducted in Table 11, and a new grouping variable has been introduced to indicate the main group to which the variables were assigned; the group was either the cannabis herb or the resin THC concentrations or daily cannabis use.

These three groups can then be summarized as indicated in Table 12, which has again been listed in descending order of median mEV. Statistical tests between these different groups using the Wilcoxson test (Supplementary Table S11) did not disclose any significant inter-group differences.

## 4. Discussion

### 4.1. Main Results

The main result of this study was the confirmation of the previously described close relationship between the various BWCAs and the metrics of cannabis exposure [4,6,7,9,10,11,12,13,14,15,92] in the modern European environment, which is characterized in many places by increasing community cannabinoid penetration [45,46]. This was conducted in both bivariate and multivariate analysis in an analytical paradigm of formal quantitative causal inference, and it also incorporated formal space–time analysis, which allows the consideration of parameters such as random effects, serial correlation, spatial correlation, and spatial autocorrelation to be formally included and accounted for in the model error structures.

### 4.2. Detailed Results

A mapping analysis showed that the incidence of body wall anomalies increased in France, Spain, and the Netherlands. Gastroschisis incidence increased in Spain, Norway, the Netherlands, and Bulgaria. Omphalocele incidence increased in Spain, the Netherlands, Norway, and Hungary. Diaphragmatic hernia incidence increased in Spain, Norway, Germany, France, and the Netherlands (Figure 3, Figure 4, Figure 5 and Figure 6).

A bivariate mapping analysis showed that body wall anomalies and the THC concentration of cannabis resin were noted to rise simultaneously in France, the Netherlands, Bulgaria, Sweden, and Norway. When the bivariate relationship of gastroschisis with cannabis resin THC concentration was considered, the two covariates were noted to rise simultaneously in Norway, Bulgaria, and the Netherlands. When the bivariate relationship of omphalocele with cannabis resin THC concentration was considered, the two covariates were noted to rise simultaneously in Norway, Sweden, France, Bulgaria, and the Netherlands. When the bivariate relationship of diaphragmatic hernia with cannabis resin THC concentration was considered, the two covariates were noted to rise simultaneously in Norway, Sweden, France, Bulgaria, and the Netherlands (Figure 8, Figure 9, Figure 10 and Figure 11).

In the bivariate analysis, whilst alcohol and tobacco were not related to the incidence of BWCAs, the amphetamine, cocaine, and cannabis resin THC content were strongly and positively related to most of these anomalies: amphetamine to abdominal wall defects and gastroschisis and cocaine to diaphragmatic hernia and omphalocele (Figure 1 and Figure 2). This effect of psychostimulants is consistent with their vasoconstrictive effects. The ranking of the BWCAs in the bivariate analysis with the cannabis metrics by median mEV was omphalocele (3.64) > diaphragmatic hernia (1.96) > abdominal wall defects (1.88) > gastroschisis (1.85) (Table 1).

In the inverse probability weighted multivariable panel regression, the series of BWCAs, the gastroschisis, omphalocele, and diaphragmatic hernia, was positively related to the various metrics of cannabis use from *p* = 2.45 × 10^−14^, 4.77 × 10^−7^ and <2.2 × 10^−16^. In the geospatial regression, the same series of BWCAs was related to cannabis metrics from *p* = 0.0016, 5.28 × 10^−6^ and 4.88 × 10^−9^ (Table 2, Table 3 and Table 4).

Seventeen out of twenty-eight (60.7%) E-value estimates were in the high range [90], as were 14/28 (50.0%) of the mEVs (Table 5, Table 6, Table 7 and Table 8). All E-value estimates and mEVs exceeded 1.25, which is the usually quoted threshold for causality.

Judging by the median mEV, the degree of association of the BWCAs with the cannabis metrics was omphalocele (31.62) > gastroschisis (9.47) > diaphragmatic hernia (8.56) (Table 10). Based on the mEVs, the order of strength of association of primary cannabis covariates was daily > cannabis herb THC concentration > cannabis resin THC concentration (Table 12), though these differences were not statistically significant.

### 4.3. Qualitative Causal Inference

The formal criteria for assigning a causal relationship to an association were set out by Hill in 1965. His nine criteria were strength of association, consistency amongst studies, specificity, temporality, coherence with known data, biological plausibility, a dose-response curve, analogy with similar situations elsewhere, and experimental confirmation. Clearly, the presently reported results elegantly fulfil these criteria.

### 4.4. Quantitative Causal Inference

One of the classical criticisms of observational studies is that the experimental groups are not truly comparable. This issue is addressed in the present work by the use of inverse probability weighting, which has the effect of transforming the analysis from an observational series only into a pseudorandomized paradigm, from which it is indeed appropriate to draw causal inferences.

The other major criticism relates to the possibility that some external and unidentified confounding variable might explain away and obviate an association which looks apparently causal on its face. This issue is addressed by the calculation of the E-value, which estimates the degree of association required of some hypothetical covariate with both the exposure of concern and the outcome of interest to obviate the observed effects. As the E-values noted in this report are largely in the moderate to higher zone, this possibility can be discounted. Confidence can additionally be drawn as the results are strongly concordant with a robust pre-existing literature.

### 4.5. Mechanisms

As outlined in the introduction, the mechanisms of cannabis genotoxicity are many and complex and have been reviewed elsewhere [4,5,6,7,16,34,35,36,37,38,39,40,41,42,93,94,95,96,97,98,99,100,101,102,103,104,105,106,107,108]. For our present purposes, we wish to focus on two pathways of particular importance, these being the disruption of key morphogen gradients and the epigenomic perturbations.

### 4.6. Cannabinoid Inhibition of Morphogens

Embryological morphogenesis is controlled to a large extent by the complex and interwoven patterning of key morphogens which control with great precision the cell movement and specification and connection formation. Disruption of these morphogenic gradients can therefore result in very serious outcomes for embryonic formation. Cannabis disrupts the signaling of most of the major morphogen gradients involved in body pattern and organ formation, including sonic hedgehog [109], retinoic acid signaling [110,111,112], notch signaling [113,114,115,116,117], Wnt signaling [118,119,120,121,122,123], and fibroblast growth factor (FGF) [124,125], including transactivation of the FGF1R by CB1R [126], bone morphogenetic proteins [127,128,129], and the hippo pathway [41].

Cannabinoids can act on these pathways either directly [109] or epigenomically, as has been demonstrated in several recent studies [40,41,42]. Epigenomic interference with hippo signaling has been demonstrated [41], as has interference with the key morphogen sonic hedgehog through at least four genes, BMP4, TMEM107, GLI3, and MEGF8 [40].

### 4.7. Epigenomic Control of Genes Relevant to Body Wall Development

One fascinating and important recent study conducted a genome-wide screen of both cannabis dependence and cannabis withdrawal and was able to provide functional annotations from Ingenuity Pathway Analysis [40]. Many findings were relevant to the present enquiry.

There was a functional annotation for body trunk development (50 genes, page 317, *p* = 0.00145 in cannabis dependence) and also in cannabis withdrawal (26 genes, *p* = 0.000555, page 340).

Body axis development was identified and classed under embryonic and organismal development (50 genes, page 302, *p* = 0.0000781, cannabis dependence).

Myogenesis was identified in this screen, including myogenesis of germ cell tumor lines (2 genes, NKX2-5 and WNT3A, page 320, *p* = 0.00177, cannabis dependence) and myogenesis of carcinoma cell lines (2 genes, NKX2-5 and WNT3A, page 320, *p* = 0.00177).

Morphogenesis of embryonic tissue was identified (page 300, 12 genes, cannabis dependence, *p* = 0.000036).

Microtubular dynamics was identified (58 genes, page 300, cannabis dependence, *p* = 0.0000333).

Cellular homeostasis was identified (73 genes, page 300, cannabis dependence, *p* = 0.0000337).

Proliferation of epithelial cells was identified (123 genes, page 300, cannabis dependence, *p* = 0.0000358).

Under the category of gene expression, DNA transcription was identified (60 genes, page 341, *p* = 0.00765, cannabis dependence). DNA recombination was identified under DNA replication, recombination, and repair (12 genes, *p* = 0.00153, page 317, cannabis dependence). Indeed, there were 256 functional annotations for DNA metabolism identified in the screen overall.

Vascular development is also highly relevant to the issue of gastroschisis as this anomaly has been suggested to have a vasculogenic basis from the observation that many vasoconstrictive drugs have been identified as causes of this anomaly [15,18,19]. It is therefore highly relevant that under angiogenesis (54 genes, *p* = 1.73 × 10^−6^, page 289, cannabis dependence) sprouting angiogenesis was identified (7 genes, page 324, cannabis dependence), and abnormal morphology of the cardiovascular system (35 genes, page 324, *p* = 0.00274) was identified.

Vasculogenesis was identified (42 genes, *p* = 0.0000665, page 302, cannabis dependence), as was movement of the vascular endothelial cells (13 genes, page 316, *p* = 0.00121, cannabis dependence; 19 genes, page 317, *p* = 0.00145; 12 genes, *p* = 0.00153, page 317).

### 4.8. Generalizability

As this study is based upon one of the world’s largest and most comprehensive databases, it uses advanced analytical techniques, including the formal tools of causal inference; it is in accordance with many other reported series, and has a strong mechanistic underpinning, including recent epigenomic data, and we are confident that these results are widely generalizable.

## 5. Strengths and Limitations

This study has a number of strengths and limitations. Its strengths include the use of one of the largest databases in the world for the BWCARs from Eurocat and the availability of the very rich EMCDDA database for drug use prevalence. The study used advanced statistical analytical techniques, including the use of inverse probability weighting, formal geospatial modelling, random forest regression for covariate selection, and the techniques of formal quantitative causal inference to analyze the data. Multi-paneled maps and graphs allowed the simultaneous presentation of time series data at a single glance. Bivariate maps were also used. Moreover, the pathophysiological framework presented to underpin the reported epidemiological results is relatively sophisticated. The limitations include those in common with many other epidemiological studies, the non-availability of individual participant cannabis exposure or BWCAR data. These limitations are common to large population epidemiological studies. Moreover, some of the covariates, especially for daily cannabis use, were missing and had to be interpolated; so, this limitation should be borne in mind when interpreting the results.

## 6. Conclusions

In summary, this study confirmed many earlier reports linking BWCARs with community cannabinoid exposure in bivariate analyses, in causal inferential multivariable paradigms, and in formal space–time analyses. Particular concern is expressed as many parts of Europe move into higher community cannabinoid penetration paradigms characterized by increased prevalence and daily intensity of the use and THC potency of the product used. The concern is that this relatively abrupt launching of parts of the continent into higher cannabinoid exposure zones will continue to see an increasing number of severe genotoxic outcomes such as those described above for amelia. Particularly when this report is read in conjunction with other reports on cannabinoid teratogenicity [4,5,6,7,11,16,108,130,131], cannabinoid cancerogenicity [95,96,97,99,100,102,103,104,106,108], cannabinoid accelerated aging [102,132,133], heritable mutagenic and carcinogenic disease [98,99,100,108,134,135,136], and heritable neurotoxicity [107,137,138,139,140,141,142,143,144,145,146], it becomes clear that rational policies in this area would tightly restrict and control community exposure to genotoxic and neurotoxic cannabinoids for multiple public health indications as has always been the community’s response to known serious genotoxic xenobiotics. The prospect of continued contamination of the food chain and increasing population exposure, incurring avoidable genetic and epigenetic damage to the heritable material of the population for multiple generations to come, is most serious indeed.

## Figures and Tables

**Table 1:** Significant Positive Slopes of Anomaly by Substance Regression Lines.

Anomaly	Substance	Mean Anomaly Rate	Estimate	Std. Error	Sigma	t_Statistic	*p*-Value	E-Value Estimate	E-Value Lower Bound
Abdominal Wall Defx	Resin	5.5353	2.7392	0.5709	0.5132	4.7978	5.33 × 10^−6^	256.74	35.01
Omphalocele	Resin	2.7816	2.7388	0.7248	0.6516	3.7785	2.62 × 10^−4^	91.17	12.13
Omphalocele	Herb	2.7816	4.7907	1.7523	0.6813	2.7340	0.0072	1201.30	11.83
Omphalocele	LMCannabis_Resin	2.7816	1.6382	0.5575	0.6676	2.9384	0.0041	18.14	3.64
Abdominal Wall Defx	Herb	5.5353	3.3920	1.5215	0.5916	2.2294	0.0276	368.45	3.20
Gastroschisis	Resin	2.2386	1.7497	0.7518	0.6759	2.3273	0.0219	20.58	2.27
Diaphragmatic Hernia	LM.Cannabis_x_Herb.THC_x_Daily.Interpol.	2.4082	1.7433	0.7672	0.6550	2.2723	0.0250	22.03	2.15
Diaphragmatic Hernia	Daily.Interpol.	2.4082	0.9085	0.3604	0.6744	2.5208	0.0133	6.27	1.96
Abdominal Wall Defx	Log(Amphetamine)	5.5353	0.2919	0.0709	0.5651	4.1184	7.04 × 10^−5^	2.58	1.88
Omphalocele	LMCannabis_Herb	2.7816	4.8916	2.4053	0.6905	2.0337	0.0442	1261.19	1.88
Gastroschisis	Log(Amphetamine)	2.2386	0.3202	0.0795	0.6341	4.0274	9.93 × 10^−5^	2.54	1.85
Omphalocele	Cocaine	2.7816	0.2500	0.0844	0.6779	2.9621	0.0037	2.15	1.49
Diaphragmatic Hernia	Cocaine	2.4082	0.2261	0.0790	0.6344	2.8624	0.0050	2.11	1.45
Abdominal Wall Defx	LMCannabis_Resin	5.5353	0.9464	0.4642	0.5558	2.0388	0.0440	8.89	1.33

**Table 2:** Geospatiotemporal Models for Gastroschisis.

Parameter Values			Model Parameters		
Parameter	Estimate (C.I.)	*p*-Value	Parameter	Value	Significance
***Additive***					
*Rate~Tobacco + Alcohol + LM.Cannabis_x_Resin.THC + Daily.Interpol. + LM.Cannabis_x_Resin.THC_x_Daily.Interpol. + LM.Cannabis_x_Herb.THC_x_Daily.Interpol. + Amphetamines + Cocaine + Income*					
Tobacco	0.06 (0.03, 0.09)	0.0003	rho	0.4379	0.0111
Daily.Interpol.	−37 (−55.44, −18.56)	8.58 × 10^−5^	lambda	−0.2487	0.141
LM.Cannabis_x_Resin.THC_x_Daily.Interpol.	1.26 (0.38, 2.14)	0.0052			
Amphetamines	0.26 (0.1, 0.41)	0.0010			
Income	0 (0, 0)	1.88 × 10^−6^			
***Interactive***					
Rate~Tobacco * Daily.Interpol. + LM.Cannabis_x_Resin.THC_x_Daily.Interpol. + LM.Cannabis_x_Herb.THC_x_Daily.Interpol. + LM.Cannabis_x_Resin.THC + Alcohol + Amphetamines + Cocaine + Income					
Tobacco	0.08 (0.04, 0.11)	5.33 × 10^−6^	rho	0.4924	0.000429
LM.Cannabis_x_Herb.THC_x_Daily.Interpol.	4.36 (1.66, 7.06)	0.0016	lambda	−0.3162	0.0265
Amphetamines	0.28 (0.13, 0.43)	0.0003			
Income	0 (0, 0)	4.39 × 10^−8^			
Tobacco: Daily.Interpol.	−2.28 (−3.34, −1.22)	2.51 × 10^−5^			
***2 Lags***					
Rate~Tobacco * Daily.Interpol. + LM.Cannabis_x_Resin.THC_x_Daily.Interpol. + LM.Cannabis_x_Resin.THC + LM.Cannabis_x_Herb.THC_x_Daily.Interpol. + Alcohol + Amphetamines + Cocaine + Income					
Tobacco	0.16 (0.1, 0.23)	4.54 × 10^−7^	rho	0.01062	0.975
Daily.Interpol.	182 (35.2, 328.8)	0.0153	lambda	−0.1374	0.642
Alcohol	−0.12 (−0.21, −0.03)	0.0078			
Amphetamines	0.21 (0.02, 0.41)	0.0351			
Cocaine	−0.59 (−1.04, −0.14)	0.0102			
Income	0 (0, 0)	4.09 × 10^−8^			
Tobacco: Daily. Interpol.	−6.77 (−12.22, −1.32)	0.0148			

**Table 3:** Geospatiotemporal Models for Omphalocele.

Parameter Values			Model Parameters		
Parameter	Estimate (C.I.)	*p*-Value	Parameter	Value	Significance
***Additive***					
*Rate~Tobacco + Alcohol + LM.Cannabis_x_Resin.THC + Resin + LM.Cannabis_x_Resin.THC_x_Daily.Interpol. + LM.Cannabis_x_Herb.THC_x_Daily.Interpol. + Amphetamines + Cocaine + Income*					
Alcohol	0.1 (0.05, 0.15)	5.28 × 10^−5^	rho	0.71867	<2 × 10^−16^
LM.Cannabis_x_Resin.THC	2.74 (1.56, 3.92)	5.28 × 10^−6^	lambda	−0.7124	2.51 × 10^−16^
LM.Cannabis_x_Herb.THC_x_Daily.Interpol.	−2.58 (−4.31, −0.85)	0.0034			
Amphetamines	−0.14 (−0.26, −0.02)	0.0258			
Cocaine	0.35 (0.16, 0.54)	0.0003			
Income	0 (0, 0)	0.0001			
***Interactive***					
Rate~Tobacco + LM.Cannabis_x_Resin.THC * Resin + LM.Cannabis_x_Resin.THC_x_Daily.Interpol. + LM.Cannabis_x_Herb.THC_x_Daily.Interpol. + Alcohol + Amphetamines + Cocaine + Income					
Tobacco	0.06 (0.03, 0.09)	1.34 × 10^−5^	rho	−0.69372	7.36 × 10^−13^
LM.Cannabis_x_Resin.THC	0.81 (0.29, 1.34)	0.0024	lambda	0.59539	1.32 × 10^−9^
Income	0 (0, 0)	1.92 × 10^−12^			
***2 Lags***					
Rate~Tobacco + Resin * LM.Cannabis_x_Resin.THC + LM.Cannabis_x_Resin.THC_x_Daily.Interpol. + LM.Cannabis_x_Herb.THC_x_Daily.Interpol. + Alcohol + Amphetamines + Cocaine + Income					
Tobacco	0.04 (0.01, 0.07)	0.0176	rho	−0.75365	2.49 × 10^−16^
Resin	−5.02 (−7.55, −2.49)	9.96 × 10^−5^	lambda	0.5649	1.03 × 10^−7^
LM.Cannabis_x_Resin.THC	6.42 (3.66, 9.18)	5.36 × 10^−6^			
LM.Cannabis_x_Herb.THC_x_Daily.Interpol.	−5.34 (−8.44, −2.24)	0.0007			
Alcohol	0.07 (0.01, 0.13)	0.0164			
Income	0 (0, 0)	5.50 × 10^−11^			

**Table 4:** Geospatiotemporal Models for Diaphragmatic Hernia.

Parameter Values			Model Parameters		
Parameter	Estimate (C.I.)	*p*-Value	Parameter	Value	Significance
***Additive***					
*Rate~Tobacco + Alcohol + LM.Cannabis + Resin + LM.Cannabis_x_Resin.THC_x_Daily.Interpol. + LM.Cannabis_x_Herb.THC_x_Daily.Interpol. + Amphetamines + Cocaine + Income*					
LM.Cannabis_x_Resin.THC_x_Daily.Interpol.	0.89 (0.39, 1.4)	0.0006	rho	0.5077	0.000167
Income	0 (0, 0)	6.01 × 10^−7^	lambda	−0.6497	2.38 × 10^−8^
***Interactive***					
Rate~Tobacco + LM.Cannabis_x_Resin.THC * Resin + LM.Cannabis_x_Resin.THC_x_Daily.Interpol. + LM.Cannabis_x_Herb.THC_x_Daily.Interpol. + Alcohol + Amphetamines + Cocaine + Income					
LM.Cannabis_x_Resin.THC	3.18 (2.12, 4.24)	4.88 × 10^−9^	rho	0.6876	<2.2 × 10^−16^
Resin	−2.7 (−3.9, −1.5)	1.03 × 10^−5^	lambda	−0.80139	<2.2 × 10^−16^
Amphetamines	−0.13 (−0.23, −0.03)	0.0086			
Income	0 (0, 0)	1.41 × 10^−8^			
***2 Lags***					
Rate~Tobacco + Resin * LM.Cannabis_x_Resin.THC_x_Daily.Interpol. + Resin + LM.Cannabis_x_Herb.THC_x_Daily.Interpol. + Alcohol + Amphetamines + Cocaine + Income					
LM.Cannabis_x_Resin.THC_x_Daily.Interpol.	1.61 (0.72, 2.5)	0.0004	rho	0.391	0.103
Income	0 (0, 0)	7.32 × 10^−5^	lambda	−0.4411	0.0539

**Table 5:** E-Values from IPW Panel Models.

Anomaly	Model and Term	*p*-Value	E-Value Estimate	Lower Bound E-Value
Gastroschisis	***Additive***			
	LM.Cannabis_x_Resin.THC_x_Daily.Interpol.	2.45 × 10^−14^	34.18	17.78
	***Interactive***			
	LM.Cannabis_x_Resin.THC_x_Daily.Interpol.	0.0006	216.81	15.7
	Tobacco: LM.Cannabis_x_Herb.THC_x_Daily.Interpol.	0.0043	1.78	1.35
	Tobacco: LM.Cannabis_x_Resin.THC	2.01 × 10^−6^	4.51	2.92
	LM.Cannabis_x_Herb.THC_x_Daily.Interpol.: LM.Cannabis_x_Resin.THC	7.50 × 10^−5^	2.11 × 10^63^	2.14 × 10^60^
	***1 Lag***			
	Tobacco: LM.Cannabis_x_Herb.THC_x_Daily.Interpol.	9.54 × 10^−6^	2.46	1.88
	Tobacco: LM.Cannabis_x_Resin.THC	0.0002	1.91	1.53
	LM.Cannabis_x_Herb.THC_x_Daily.Interpol.: LM.Cannabis_x_Resin.THC	9.28 × 10^−6^	7.52 × 10^95^	8.16 × 10^55^
	***2 Lags***			
	Tobacco: LM.Cannabis_x_Herb.THC_x_Daily.Interpol.	0.0097	1.7	1.27
	Tobacco: LM.Cannabis_x_Resin.THC	0.0003	6.75	3.13
	LM.Cannabis_x_Herb.THC_x_Daily.Interpol.: LM.Cannabis_x_Resin.THC	9.78 × 10^−11^	1.86 × 10^16^	9.25 × 10^11^
Omphalocele	***Additive***			
	LM.Cannabis_x_Resin.THC	4.77 × 10^−7^	5.77	3.56
	***Interactive***			
	LM.Cannabis_x_Resin.THC	0.0059	4.10 × 10^5^	80.95
	***2 Lags***			
	LM.Cannabis_x_Resin.THC	2.30 × 10^−6^	7.04	3.95
Diaphragmatic Hernia	***Additive***			
	LM.Cannabis_x_Resin.THC_x_Daily.Interpol.	0.0162	3.38	1.55
	LM.Cannabis	0.0044	7.92 × 10^7^	600.88
	***Interactive***			
	LM.Cannabis_x_Herb.THC	5.96 × 10^−5^	7.48 × 10^21^	5.55 × 10^11^
	Tobacco: LM.Cannabis_x_Herb.THC_x_Daily.Interpol.: LM.Cannabis_x_Herb.THC	0.0281	3.67	1.41
	***2 Lags***			
	LM.Cannabis_x_Resin.THC_x_Daily.Interpol.	<2.2 × 10^−16^	16.79	10.92

**Table 6:** E-Values from Geospatial Models.

Anomaly	Model and Term	*p*-Value	E-Value Estimate	Lower Bound E-Value
Gastroschisis	***Additive***			
	LM.Cannabis_x_Resin.THC_x_Daily.Interpol.	0.0052	17.08	3.24
	***Interactive***			
	LM.Cannabis_x_Herb.THC_x_Daily.Interpol.	0.0016	4.07 × 10^3^	35.65
	***2 Lags***			
	Daily.Interpol.	0.0153	Infinity	9.23 × 10^44^
Omphalocele	***Additive***			
	LM.Cannabis_x_Resin.THC	5.28 × 10^−6^	772.14	59.28
	***Interactive***			
	LM.Cannabis_x_Resin.THC	0.0024	8.41	2.80
	***2 Lags***			
	LM.Cannabis_x_Resin.THC	5.36 × 10^−6^	9.84 × 10^5^	3.51 × 10^3^
Diaphragmatic Hernia	***Additive***			
	LM.Cannabis_x_Resin.THC_x_Daily.Interpol.	0.0006	10.29	3.57
	***Interactive***			
	LM.Cannabis_x_Resin.THC	4.88 × 10^−9^	2.54 × 10^3^	232.68
	***2 Lags***			
	LM.Cannabis_x_Resin.THC_x_Daily.Interpol.	0.0004	30.22	6.19

**Table 7:** All E-Values.

No.	Anomaly	Regression	Model Type	Term	Group	*p*-Value	E-Value Estimate	Lower Bound E-Value
1	Gastroschisis	Panel	Interactive	LM.Cannabis_x_Herb.THC_x_Daily.Interpol.: LM.Cannabis_x_Resin.THC	Herb	7.50 × 10^−5^	2.11 × 10^63^	2.14 × 10^60^
2	Gastroschisis	Panel	1 Lag	LM.Cannabis_x_Herb.THC_x_Daily.Interpol.: LM.Cannabis_x_Resin.THC	Herb	9.28 × 10^−6^	7.52 × 10^95^	8.16 × 10^55^
3	Gastroschisis	Spatial	2 Lags	Daily.Interpol.	Daily	0.0153	Infinity	9.23 × 10^44^
4	Diaphragmatic Hernia	Panel	Additive	LM.Cannabis	Herb	0.0044	7.92 × 10^7^	600.88
5	Diaphragmatic Hernia	Panel	Interactive	LM.Cannabis_x_Herb.THC	Herb	5.96 × 10^−5^	7.48 × 10^21^	5.55 × 10^11^
6	Gastroschisis	Spatial	Interactive	LM.Cannabis_x_Herb.THC_x_Daily.Interpol.	Herb	0.0016	4.07 × 10^3^	35.65
7	Gastroschisis	Panel	2 Lags	LM.Cannabis_x_Herb.THC_x_Daily.Interpol.: LM.Cannabis_x_Resin.THC	Herb	9.78 × 10^−11^	1.86 × 10^16^	9.25 × 10^11^
8	Omphalocele	Spatial	2 Lags	LM.Cannabis_x_Resin.THC	Resin	5.36 × 10^−6^	9.84 × 10^5^	3.51 × 10^3^
9	Diaphragmatic Hernia	Spatial	Interactive	LM.Cannabis_x_Resin.THC	Resin	4.88 × 10^−9^	2.54 × 10^3^	232.68
10	Omphalocele	Panel	Interactive	LM.Cannabis_x_Resin.THC	Resin	0.0059	4.10 × 10^5^	80.95
11	Omphalocele	Spatial	Additive	LM.Cannabis_x_Resin.THC	Resin	5.28 × 10^−6^	772.14	59.28
12	Omphalocele	Panel	2 Lags	LM.Cannabis_x_Resin.THC	Resin	2.30 × 10^−6^	7.04	3.95
13	Omphalocele	Panel	Additive	LM.Cannabis_x_Resin.THC	Resin	4.77 × 10^−7^	5.77	3.56
14	Omphalocele	Spatial	Interactive	LM.Cannabis_x_Resin.THC	Resin	0.0024	8.41	2.80
15	Gastroschisis	Panel	Additive	LM.Cannabis_x_Resin.THC_x_Daily.Interpol.	Resin	2.45 × 10^−14^	34.18	17.78
16	Gastroschisis	Panel	Interactive	LM.Cannabis_x_Resin.THC_x_Daily.Interpol.	Resin	0.0006	216.81	15.7
17	Diaphragmatic Hernia	Panel	2 Lags	LM.Cannabis_x_Resin.THC_x_Daily.Interpol.	Resin	<2.2 × 10^−16^	16.79	10.92
18	Diaphragmatic Hernia	Spatial	2 Lags	LM.Cannabis_x_Resin.THC_x_Daily.Interpol.	Resin	0.0004	30.22	6.19
19	Diaphragmatic Hernia	Spatial	Additive	LM.Cannabis_x_Resin.THC_x_Daily.Interpol.	Resin	0.0006	10.29	3.57
20	Gastroschisis	Spatial	Additive	LM.Cannabis_x_Resin.THC_x_Daily.Interpol.	Resin	0.0052	17.08	3.24
21	Diaphragmatic Hernia	Panel	Additive	LM.Cannabis_x_Resin.THC_x_Daily.Interpol.	Resin	0.0162	3.38	1.55
22	Gastroschisis	Panel	1 Lag	Tobacco: LM.Cannabis_x_Herb.THC_x_Daily.Interpol.	Herb	9.54 × 10^−6^	2.46	1.88
23	Gastroschisis	Panel	Interactive	Tobacco: LM.Cannabis_x_Herb.THC_x_Daily.Interpol.	Herb	0.0043	1.78	1.35
24	Gastroschisis	Panel	2 Lags	Tobacco: LM.Cannabis_x_Herb.THC_x_Daily.Interpol.	Herb	0.0097	1.7	1.27
25	Diaphragmatic Hernia	Panel	Interactive	Tobacco: LM.Cannabis_x_Herb.THC_x_Daily.Interpol.: LM.Cannabis_x_Herb.THC	Herb	0.0281	3.67	1.41
26	Gastroschisis	Panel	2 Lags	Tobacco: LM.Cannabis_x_Resin.THC	Resin	0.0003	6.75	3.13
27	Gastroschisis	Panel	Interactive	Tobacco: LM.Cannabis_x_Resin.THC	Resin	2.01 × 10^−6^	4.51	2.92
28	Gastroschisis	Panel	1 Lag	Tobacco: LM.Cannabis_x_Resin.THC	Resin	0.0002	1.91	1.53

**Table 8:** Sequential List of E-Values.

No.	E-Value Estimate	Lower Bound E-Value
1	Infinity	2.14 × 10^60^
2	7.52 × 10^95^	8.16 × 10^55^
3	2.11 × 10^63^	9.23 × 10^44^
4	7.48 × 10^21^	9.25 × 10^11^
5	1.86 × 10^16^	5.55 × 10^11^
6	7.92 × 10^7^	3.51 × 10^3^
7	9.84 × 10^5^	600.88
8	4.10 × 10^5^	232.68
9	4.07 × 10^3^	80.95
10	2.54 × 10^3^	59.28
11	772.14	35.65
12	216.81	17.78
13	34.18	15.7
14	30.22	10.92
15	17.08	6.19
16	16.79	3.95
17	10.29	3.57
18	8.41	3.56
19	7.04	3.24
20	6.75	3.13
21	5.77	2.92
22	4.51	2.80
23	3.67	1.88
24	3.38	1.55
25	2.46	1.53
26	1.91	1.41
27	1.78	1.35
28	1.7	1.27

**Table 9:** E-Values by Anomaly.

No.	Anomaly	Regression	Model Type	Term	Group	*p*-Value	E-Value Estimate	Lower Bound E-Value
1	Diaphragmatic Hernia	Panel	Additive	LM.Cannabis	Herb	0.0044	7.92 × 10^7^	600.88
2	Diaphragmatic Hernia	Panel	Interactive	LM.Cannabis_x_Herb.THC	Herb	5.96 × 10^−5^	7.48 × 10^21^	5.55 × 10^11^
3	Diaphragmatic Hernia	Spatial	Interactive	LM.Cannabis_x_Resin.THC	Resin	4.88 × 10^−9^	2.54 × 10^3^	232.68
4	Diaphragmatic Hernia	Panel	2 Lags	LM.Cannabis_x_Resin.THC_x_Daily.Interpol.	Resin	<2.2 × 10^−16^	16.79	10.92
5	Diaphragmatic Hernia	Spatial	2 Lags	LM.Cannabis_x_Resin.THC_x_Daily.Interpol.	Resin	0.0004	30.22	6.19
6	Diaphragmatic Hernia	Spatial	Additive	LM.Cannabis_x_Resin.THC_x_Daily.Interpol.	Resin	0.0006	10.29	3.57
7	Diaphragmatic Hernia	Panel	Additive	LM.Cannabis_x_Resin.THC_x_Daily.Interpol.	Resin	0.0162	3.38	1.55
8	Diaphragmatic Hernia	Panel	Interactive	Tobacco: LM.Cannabis_x_Herb.THC_x_Daily.Interpol.: LM.Cannabis_x_Herb.THC	Herb	0.0281	3.67	1.41
9	Gastroschisis	Panel	Interactive	LM.Cannabis_x_Herb.THC_x_Daily.Interpol.: LM.Cannabis_x_Resin.THC	Herb	7.50 × 10^−5^	2.11 × 10^63^	2.14 × 10^60^
10	Gastroschisis	Panel	1 Lag	LM.Cannabis_x_Herb.THC_x_Daily.Interpol.: LM.Cannabis_x_Resin.THC	Herb	9.28 × 10^−6^	7.52 × 10^95^	8.16 × 10^55^
11	Gastroschisis	Spatial	2 Lags	Daily.Interpol.	Daily	0.0153	Infinity	9.23 × 10^44^
12	Gastroschisis	Spatial	Interactive	LM.Cannabis_x_Herb.THC_x_Daily.Interpol.	Herb	0.0016	4.07 × 10^3^	35.65
13	Gastroschisis	Panel	2 Lags	LM.Cannabis_x_Herb.THC_x_Daily.Interpol.: LM.Cannabis_x_Resin.THC	Herb	9.78 × 10^−11^	1.86 × 10^16^	9.25 × 10^11^
14	Gastroschisis	Panel	Additive	LM.Cannabis_x_Resin.THC_x_Daily.Interpol.	Resin	2.45 × 10^−14^	34.18	17.78
15	Gastroschisis	Panel	Interactive	LM.Cannabis_x_Resin.THC_x_Daily.Interpol.	Resin	0.0006	216.81	15.7
16	Gastroschisis	Spatial	Additive	LM.Cannabis_x_Resin.THC_x_Daily.Interpol.	Resin	0.0052	17.08	3.24
17	Gastroschisis	Panel	1 Lag	Tobacco: LM.Cannabis_x_Herb.THC_x_Daily.Interpol.	Herb	9.54 × 10^−6^	2.46	1.88
18	Gastroschisis	Panel	Interactive	Tobacco: LM.Cannabis_x_Herb.THC_x_Daily.Interpol.	Herb	0.0043	1.78	1.35
19	Gastroschisis	Panel	2 Lags	Tobacco: LM.Cannabis_x_Herb.THC_x_Daily.Interpol.	Herb	0.0097	1.7	1.27
20	Gastroschisis	Panel	2 Lags	Tobacco: LM.Cannabis_x_Resin.THC	Resin	0.0003	6.75	3.13
21	Gastroschisis	Panel	Interactive	Tobacco: LM.Cannabis_x_Resin.THC	Resin	2.01 × 10^−6^	4.51	2.92
22	Gastroschisis	Panel	1 Lag	Tobacco: LM.Cannabis_x_Resin.THC	Resin	0.0002	1.91	1.53
23	Omphalocele	Spatial	2 Lags	LM.Cannabis_x_Resin.THC	Resin	5.36 × 10^−6^	9.84 × 10^5^	3.51 × 10^3^
24	Omphalocele	Panel	Interactive	LM.Cannabis_x_Resin.THC	Resin	0.0059	4.10 × 10^5^	80.95
25	Omphalocele	Spatial	Additive	LM.Cannabis_x_Resin.THC	Resin	5.28 × 10^−6^	772.14	59.28
26	Omphalocele	Panel	2 Lags	LM.Cannabis_x_Resin.THC	Resin	2.30 × 10^−6^	7.04	3.95
27	Omphalocele	Panel	Additive	LM.Cannabis_x_Resin.THC	Resin	4.77 × 10^−7^	5.77	3.56
28	Omphalocele	Spatial	Interactive	LM.Cannabis_x_Resin.THC	Resin	0.0024	8.41	2.80

**Table 10:** Summary of E-Values by Anomaly.

Anomaly	Number	Mean Minimum E-Value	Median Minimum E-Value	Min Minimum E-Value	Max Minimum E-Value	Mean E-Value Estimate	Median E-Value Estimate	Min E-Value Estimate	Max E-Value Estimate
Omphalocele	6	610.09	31.62	2.80	3510	2.32 × 10^5^	390.28	5.77	9.84 × 10^5^
Gastroschisis	14	1.53 × 10^59^	9.47	1.27	2.14 × 10^60^	1.07 × 10^306^	25.63	1.70	1.50 × 10^307^
Diaphragmatic Hernia	8	6.94 × 10^10^	8.56	1.41	5.55 × 10^11^	9.35 × 10^20^	23.51	3.38	7.48 × 10^21^

**Table 11:** E-Values by Group.

Anomaly	Regression	Model Type	Term	Group	*p*-Value	E-Value Estimate	Lower Bound E-Value
Gastroschisis	Panel	Interactive	LM.Cannabis_x_Herb.THC_x_Daily.Interpol.: LM.Cannabis_x_Resin.THC	Herb	7.50 × 10^−5^	2.11 × 10^63^	2.14 × 10^60^
Gastroschisis	Panel	1 Lag	LM.Cannabis_x_Herb.THC_x_Daily.Interpol.: LM.Cannabis_x_Resin.THC	Herb	9.28 × 10^−6^	7.52 × 10^95^	8.16 × 10^55^
Gastroschisis	Spatial	2 Lags	Daily.Interpol.	Daily	0.0153	Infinity	9.23 × 10^44^
Diaphragmatic Hernia	Panel	Additive	LM.Cannabis	Herb	0.0044	7.92 × 10^7^	600.88
Diaphragmatic Hernia	Panel	Interactive	LM.Cannabis_x_Herb.THC	Herb	5.96 × 10^−5^	7.48 × 10^21^	5.55 × 10^11^
Gastroschisis	Spatial	Interactive	LM.Cannabis_x_Herb.THC_x_Daily.Interpol.	Herb	0.0016	4.07 × 10^3^	35.65
Gastroschisis	Panel	2 Lags	LM.Cannabis_x_Herb.THC_x_Daily.Interpol.: LM.Cannabis_x_Resin.THC	Herb	9.78 × 10^−11^	1.86 × 10^16^	9.25 × 10^11^
Omphalocele	Spatial	2 Lags	LM.Cannabis_x_Resin.THC	Resin	5.36 × 10^−6^	9.84 × 10^5^	3.51 × 10^3^
Diaphragmatic Hernia	Spatial	Interactive	LM.Cannabis_x_Resin.THC	Resin	4.88 × 10^−9^	2.54 × 10^3^	232.68
Omphalocele	Panel	Interactive	LM.Cannabis_x_Resin.THC	Resin	0.0059	4.10 × 10^5^	80.95
Omphalocele	Spatial	Additive	LM.Cannabis_x_Resin.THC	Resin	5.28 × 10^−6^	772.14	59.28
Omphalocele	Panel	2 Lags	LM.Cannabis_x_Resin.THC	Resin	2.30 × 10^−6^	7.04	3.95
Omphalocele	Panel	Additive	LM.Cannabis_x_Resin.THC	Resin	4.77 × 10^−7^	5.77	3.56
Omphalocele	Spatial	Interactive	LM.Cannabis_x_Resin.THC	Resin	0.0024	8.41	2.80
Gastroschisis	Panel	Additive	LM.Cannabis_x_Resin.THC_x_Daily.Interpol.	Resin	2.45 × 10^−14^	34.18	17.78
Gastroschisis	Panel	Interactive	LM.Cannabis_x_Resin.THC_x_Daily.Interpol.	Resin	0.0006	216.81	15.7
Diaphragmatic Hernia	Panel	2 Lags	LM.Cannabis_x_Resin.THC_x_Daily.Interpol.	Resin	<2.2 × 10^−16^	16.79	10.92
Diaphragmatic Hernia	Spatial	2 Lags	LM.Cannabis_x_Resin.THC_x_Daily.Interpol.	Resin	0.0004	30.22	6.19
Diaphragmatic Hernia	Spatial	Additive	LM.Cannabis_x_Resin.THC_x_Daily.Interpol.	Resin	0.0006	10.29	3.57
Gastroschisis	Spatial	Additive	LM.Cannabis_x_Resin.THC_x_Daily.Interpol.	Resin	0.0052	17.08	3.24
Diaphragmatic Hernia	Panel	Additive	LM.Cannabis_x_Resin.THC_x_Daily.Interpol.	Resin	0.0162	3.38	1.55
Gastroschisis	Panel	1 Lag	Tobacco: LM.Cannabis_x_Herb.THC_x_Daily.Interpol.	Herb	9.54 × 10^−6^	2.46	1.88
Gastroschisis	Panel	Interactive	Tobacco: LM.Cannabis_x_Herb.THC_x_Daily.Interpol.	Herb	0.0043	1.78	1.35
Gastroschisis	Panel	2 Lags	Tobacco: LM.Cannabis_x_Herb.THC_x_Daily.Interpol.	Herb	0.0097	1.7	1.27
Diaphragmatic Hernia	Panel	Interactive	Tobacco: LM.Cannabis_x_Herb.THC_x_Daily.Interpol.: LM.Cannabis_x_Herb.THC	Herb	0.0281	3.67	1.41
Gastroschisis	Panel	2 Lags	Tobacco: LM.Cannabis_x_Resin.THC	Resin	0.0003	6.75	3.13
Gastroschisis	Panel	Interactive	Tobacco: LM.Cannabis_x_Resin.THC	Resin	2.01 × 10^−6^	4.51	2.92
Gastroschisis	Panel	1 Lag	Tobacco: LM.Cannabis_x_Resin.THC	Resin	0.0002	1.91	1.53

**Table 12:** Summary of E-Values by Group.

Group	Number	Mean Minimum E-Value	Median Minimum E-Value	Minimum Minimum E-Value	Maximum Minimum E-Value	Mean E-Value Estimate	Median E-Value Estimate	Minimum E-Value Estimate	Maximum E-Value Estimate
Daily	6	9.23 × 10^44^	9.23 × 10^44^	9.23 × 10^44^	9.23 × 10^44^	1.50 × 10^307^	1.50 × 10^307^	1.50 × 10^307^	1.50 × 10^307^
Herb	14	2.14 × 10^59^	318.265	1.27	2.14 × 10^60^	7.52 × 10^94^	3.96 × 10^7^	1.7	7.52 × 10^95^
Resin	8	232.93	3.95	1.53	3510	82216.19	16.79	1.91	9.84 × 10^5^

## Data Availability

All data generated or analyzed during this study are included in this published article and its Supplementary Information Files. Data along with the relevant R code have been made publicly available on the Mendeley Database Repository and can be accessed from these URLs: https://doi.org/10.17632/tysn37t426.1 and https://doi.org/10.17632/vd6mt5r5jm.1 (accessed on 10 January 2022).

## Supplementary Materials

The following supporting information can be downloaded at: https://www.mdpi.com/article/10.3390/ijerph19159027/s1, Figure S1: International geospatial links used for computing the sparse spatial weights matrix for use in the geospatial regressions (A) edited and (B) final links; Table S1: Overall Study Profile; Table S2: Daily Cannabis Use—Raw Data; Table S3: Daily Cannabis Use—Interpolated Data; Table S4: All regression slopes and results from bivariate analyses; Table S5: Variable Importance Tables from Ranger random forest regression—Gastroschisis; Table S6: Variable Importance Tables from Ranger random forest regression—Omphalocele; Table S7: Variable Importance Tables from Ranger random forest regression—Diaphragmatic Hernia; Table S8: Inverse probability weighted panel regression results—Gastroschisis; Table S9: Inverse probability weighted panel regression results—Omphalocele; Table S10: Inverse probability weighted panel regression results—Diaphragmatic hernia; Table S11: Wilcoxson tests comparing main covariate groups.
