# Supplementary material for: European Epidemiological Patterns of Cannabis- and Substance-Related Body Wall Congenital Anomalies: Geospatiotemporal and Causal Inferential Study
Source: Int J Environ Res Public Health. 2022 Jul 25;19(15):9027. doi: 10.3390/ijerph19159027 (PMC9330494; doi:10.3390/ijerph19159027)
Supplement: Supplementary file 1 [file ijerph-19-09027-s001.zip › ijerph-1804252-supplementary/Figure S1 - Body Wall Spatial Links.pdf]

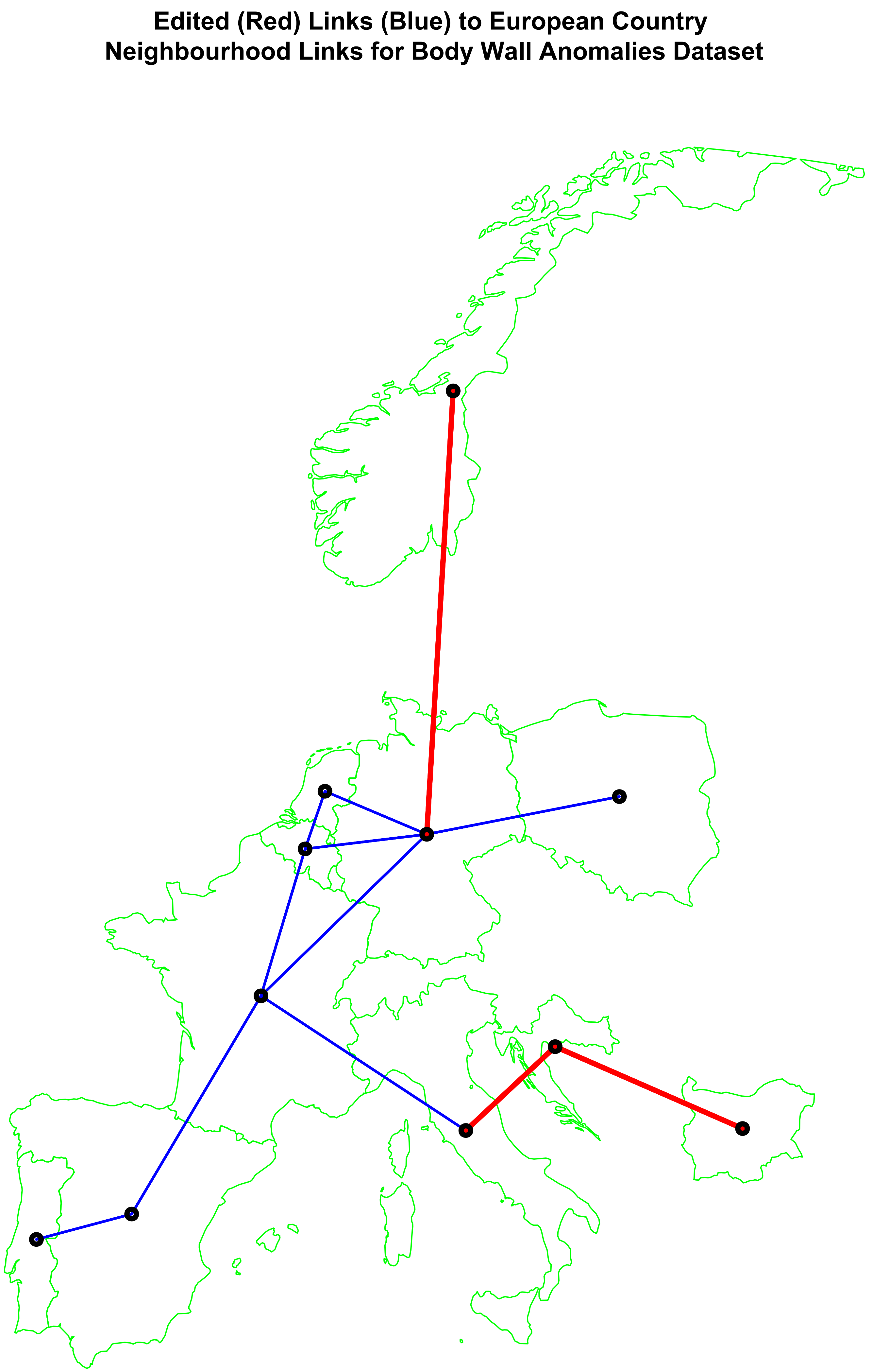
Edited (Red) Links (Blue) to European Country
Neighbourhood Links for Body Wall Anomalies Dataset

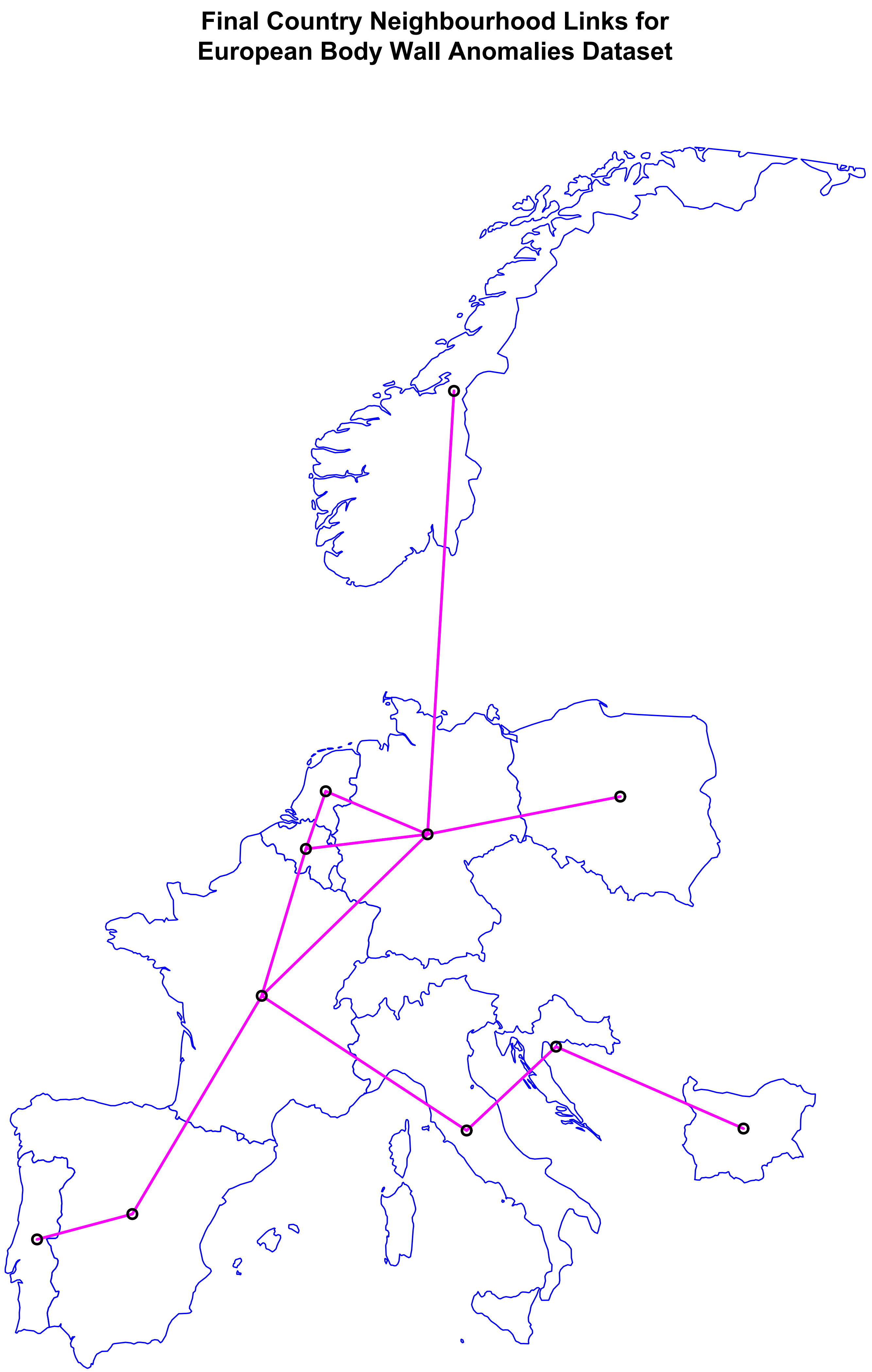
Final Country Neighbourhood Links for
European Body Wall Anomalies Dataset
